# Editorial

**Published:** 1861-02

**Authors:** 


### Editorial.

### COMMENCEMENT EXERCISES.

The Commencement exercises of the present course of the Ohio Dental College will be held in the first lecture room of the College, on Wednesday evening, Feb. 20th, at 7½ o'clock. All the members of the profession and friends of the institution are cordially invited to be present. T.

### MEETING OF THE MISS. VALLEY ASSOCIATION.

The regular annual meeting of this association will take place on Wednesday, Feb. 20th, at 10 o'clock, A. M. We hope there will be a full meeting. It always gives us much pleasure to meet with the members of this association. We are always anxious to enjoy its meetings, and always sad at the time of its adjournment. It is the oldest dental association in the world; other associations came into existence, became superannuated and died, or fulfilled their mission, and passed out of existence. The Mississippi Valley Association differs from all such, in this, that it is never weary nor exhausted in doing good work, and it does not become superannuated, for it works more vigorously now than ever before; it seems to be constantly renewing its age, and gathering strength, for the accomplishment of new purposes. We anticipate a very full meeting of the working men of the profession. T.

### ANÆSTHETICS.—BY JOHN SNOW, M. D.

This is the most complete treatise on anæsthetics extant. The author enters into a discussion of all the anæsthetic agents employed, discusses their properties most fully and the method of using them, and presents all the information pertinent to this subject, that is developed. He gives a large number and great variety of cases, tending to illustrate the method of using it in different conditions and under different circumstances.

This is a valuable work and should be in the hands of every one who administers anæsthetics. T.

### THE DENTIST'S MEMORANDUM.

This is an engagement book gotten up and arranged by Dr. C. H. Cleaveland, and published by J. T. Toland. It is more full and complete than anything we have before seen; it is arranged for appointments for each hour from 8 A. M. to 5 P. M. with space for the name of the patient, and the character of the operation; it is also ruled for dollars and cents, on a line with the name of patient; it thus serves as a day-book from which the accounts may be posted into the ledger. It contains quite a number of formulas and recipes some of which are good. There are quite a number of paragraphs on common subjects pertaining to dentistry, in which many good and useful suggestions are made in regard to conditions and treatment.

The chief value of the work consists in the admirable arrangement of the appointment department. T.

### DOES AMALGAM SHRINK?

Dr. Latimer, under the modest title of "Little Things," in the January number of the *Cosmos*, raises a pertinent inquiry in regard to the shrinkage of amalgams. He tried an experiment with one, and found that it expanded, and says, in conclusion, "This, indeed, is what I might have anticipated, from a consideration of the well-known fact that most bodies expand in the act of crystallization." He adds, farther: "I do not offer this in defense of amalgam, but as a scientific fact."

The fact is interesting and would be much more so if we knew the composition of the amalgam used. Some amalgams contract. Some expand. The fact of crystallization has but little to do in the matter. Mercury itself *contracts* in crystallizing. We like the idea of "Little Things." W.

### A CORK VISE.

"But we do not regard it as treating a patient as a *human being*, with nerves and spasmodic muscles about the mouth and face, by putting the mouth in a *vice*, as it virtually is in using a cork between the teeth."

So writes "J. D. W." in the *Dental Cosmos;* and will he, just to please us, hold his mouth, for a given time, so wide open that the grinding surfaces of the first molars will be about a half inch apart, and then after resting himself, prop them just as far apart, with a cork, for the same length of time, and tell us which fatigues him most? (We would do it for him, were the case reversed. Certainly we would.) And if he tells us he is more fatigued with, than without the cork, we will give it up—with the understanding that the muscles which open his mouth have more contractile power than those which close it. W.

### PRETTY FAST.

"Dr. Wetherbee said—the secondary dentine which is found in the teeth of old persons takes fifteen or twenty minutes to be deposited."—*Cosmos, p.* 344. O, come now! Mr. Reporter, call it *years* and see if we don't fall in with you.

### NEW DENTAL SOCIETY.

We learn that the dentists of central Ohio, recently met and formed a dental association at Columbus. This is a good move, and one that we are glad to see. The brethren of that vicinity, have, we think, been somewhat remiss in regard to the matter of association. We have met very few of them in dental associations, but we hope they will go to work in earnest and make up for past delinquencies. We have not the proceedings at hand, but hope we shall have soon. T.

